# The Life of an Army Dentist

**Published:** 1905-11-15

**Authors:** Samuel W. Hussey


### THE LIFE OF AN ARMY DENTIST.

BY SAMUEL W. HUSSEY, D.D.S.

The following extract from a contract army surgeon's report to the Surgeon General will give some idea of the duties and usefulness of these officers—EDITOR REGISTER.

I have the honor to submit the following report of my presonal services in the Departments of California and Dakota, for the fiscal year ending June 30, 1904.

July 12th to 31st, 1903, on duty with the Examining and Supervising Dental Surgeon at the post of Presidio of San Francisco August 1st to August 6th, 1903, enroute from Presidio, San Francisco, Cal., to St. Paul, Minn. August 6th, to August 10th, 1903, at St. Paul, Minn., awaiting orders for assignment to duty. August 10th to August 13th, 1903, at Ft. Snelling, Minn., August 13th and 14th, 1903, enroute from Ft. Snelling, Minn., to Ft. Lincoln, N. Dakota. August

15th, 1903, on duty at Ft. Lincoln, N. Dakota. August 16th, 1903, enroute from Ft. Lincoln N. Dakota to Ft. Yates, N. Dakota. August 17th, 1903, on duty at Ft. Yates, N. Dakota. August 18th, 1903, delayed at Ft. Yates, N. Dakota, on account of transportation. August 19th and 20th, 1903, enroute from Ft. Yates, N. Dakota, to Ft. Keogh, Mont. August 21st to 23rd, 1903, on duty at Ft. Keogh, Mont. August 23rd and 24th, 1903, enroute from Ft. Keogh, Mont., to Ft. Yellowstone, Wyo. August 24th to 27th, 1903, on duty at Ft. Yellowstone, Wyo. August 27th to 29th, 1903, enroute from Ft. Yellowstone, Wyo., to Ft. Harrison, Mont. August 29th to 31st, on duty at Ft. Harrison, Mont. September 1st and 2nd, 1903, enroute from Ft. Marrison, Mont., to Ft. Assinniboine, Mont. September 2nd to 7th, 1903, on duty at Ft. Assinniboine, Mont. September 7th, enroute from Ft. Assinniboine, Mont., to Ft. Harrison, Mont. September 8th to 10th, 1903, on duty at Ft. Harrison, Mont. September 10th, 1903, enroute from Ft. Harrison, Mont., to Ft. Missoula, Mont. September 11th and 12th, 1903, on duty at Ft. Missoula, Mont. September 12th to 15th, 1903, enroute from Ft. Missoula Mont., to Ft. Meade, S. Dakota. September 15th and 16th, on duty at Ft. Meade, S. Dakota. September 16th to 18th, 1903, enroute from Ft. Meade, S. Dakota, to Ft. Snelling, Minn. Sepetmber 19th to November 30th, 1903, on duty at Ft. Snelling, Minn. December 1st and 2nd, 1903, enroute from Ft. Snelling, Minn., to Ft. Meade, S. Dakota. December 3rd, 1903, to January 14th, 1904, on duty at Ft. Meade, S. Dakota. January 15th to 18th, 1904, enroute from Ft. Meade, S. Dakota, to Ft. Lincoln, N. Dakota. January 14th to February 5th, 1904, on duty at Ft. Lincoln, N. Dakota. February 5th, 1904, enroute from Ft. Lincoln, N. Dakota, to Ft. Keogh, Mont. February 6th to March 20th, 1904, on duty at Ft. Keogh, Mont. March 20th and 21st, 1904, enroute from Ft. Keogh Mont., to Ft. Yellowstone, Wyo. March 22nd to April 4th, 1904, on duty at Ft. Yellowstone, Wyo. April 5th to 7th, 1904, enroute from Yellowstone, Wyo., to Ft. Harrison, Mont. April 8th to May 7th, 1904, on duty at Ft. Harrison, Mont. May 7th and 8th, enroute from Ft. Harrison, Mont., to Ft. Assinniboin, Mont. May 8th to June 30th, 1904, on duty at Ft. Assinniboin, Mont.

CHARACTER OF SERVICE.

My services from July 1st to 31st, 1903, are consolidated with those of the Examining and Supervising Dental Surgeon, at the Presidio of San Francisco, a report of which will be rendered in his personal service report for 1904.

The dental service rendered in the Department of Dakota, from August 12th, 1903, to June 30th, 1904, has been almost entirely of an emergency character.

My first tour of duty from August 12th to September 18th, 1903, I visited all the posts in the Department for the purpose of examining the teeth of all the officers and enlisted men and perform operations only that required immediate attention, and upon arrival at Ft. Snelling, I reported to the Department Commander the probable length of time required to complete the necessary dental work at each post.

After remaining on duty at Ft. Snelling, Minn., from September 18th to November 30th, 1903, I was sent out on the second tour of duty to visit all the posts in the Department and perform all the necessary dental work.

On this tour my services were very largely extractions, restorations by permanent fillings of gold, amalgam and oxy-phosphate, removing of salivary deposits, and treatment of abscesses and other ordinary diseased condition of the teeth and gums.

The hours for Sundays and holidays have been found necessary for the relief of the suffering in new cases and redressing of cases already under treatment.

The office hours have been the greater part of the time from 8:30 A. M. to 5:00 P. M., except during the short days of the winter, when the light was not quite sufficient.

Total number of patients treated, 815, as follows: Officers, regular, 55; Enlisted men, 674; Retired soldiers, 1; General prisoners, 3; Civilian attaches, male, 16; Civilian attaches, female, 66. Total number of sittings, 2,200; Total number of sittings per each working day, 9; Average number of sittings per patient, 3; Average length of time required for completing treatment of each patient, one hour and twenty minutes.

### DENTAL AND ORAL DISEASES AND INJURIES.

Affecting the teeth and gums, 1524, as follows: Defective fillings, 20; Dental carries, 1,041; Dento-alveolar abscess, 84; Devitalized pulp, 93; Erosion chemical, 1; Erupting teeth, 2; Erupting teeth, painful, 1; Hypertrophy of the gums, 1; Pericementitis chronic, 1; Pulpitis acute, 32; Pulpitis chronic, 35; Pyorrhea alveolaris, 1; Resorption of the alveolar process, 7; Salivary deposits, 198; Sensitive dentine, 5; Supernumerary teeth, 2.

Affecting the mouth and jaws, 19, as follows: Fracture of the jaws, inferior, 1; Gingivitis, simple, 7; Gingivitis, ulcerative, 5; Gingivitis, syphilitic, 1; Leucoplakia, buccal, 2 Leucoplakia, lingual, 1; Neuralgia, facial, 1; Stomatitis, syphilitic, 1;.

Operations performed on the teeth and gums, 853, as follows: Abscesses lanced, 2; Excision of the gums, 2; Pulp devitalized, 30; Pulp extirpated, 60; Root canals filled with gutta-percha, 45; Salivary deposits removed, 198; Teeth extracted, 575,; Teeth treated, medicated, 181.

Angle mechanical appliance for fractured jaws, 1.

Restoration of teeth by filling, etc., 394, as follows: Amalgam, 118; Gold, 17; Gutta-percha, 2, Oxyphosphate, 198.

Restoration of teeth by combination fillings, 45, as follows: Gutta-percha and oxyphosphate, 26; Oxyphate and amalgam, 18; Gutta-percha, oxyphosphate and amalgam, 1.

Crowns set, 14, as follows: Gold shell, 4; Logan, 8; Reset, 2.

The force of dental surgeons in the Department of Dakota, is still inadequate to meet the demands of all classes of operations, *e. g.*, it has been impossible for the present dental surgeon to perform restorations by artificial dentures, except while stationed at Ft. Snelling, Minn., because it is impracticable to carry an extensive laboratory equipment on tours of duty while visiting posts from two weeks to two months and the greater demand requires treatment and filling of such a nature as has been mentioned above, therefore the laboratory outfit has been lying idle at Fort Snelling, Minn., for several months.

